# Intra‐cortical myelin mediates personality differences

**DOI:** 10.1111/jopy.12442

**Published:** 2018-11-13

**Authors:** Nicola Toschi, Luca Passamonti

**Affiliations:** ^1^ Department of Biomedicine & Prevention University “Tor Vergata” Rome Italy; ^2^ Department of Radiology Martinos Center for Biomedical Imaging Boston Massachusetts; ^3^ Institute of Bioimaging & Molecular Physiology National Research Council Milano Italy; ^4^ Department of Clinical Neurosciences University of Cambridge Cambridge UK

**Keywords:** Big‐Five, individual differences, myelin, myelination, T1/T2‐weighted ratio

## Abstract

**Objective:**

Differences in myelination in the cortical mantle are important neurobiological mediators of variability in cognitive, emotional, and behavioral functioning. Past studies have found that personality traits reflecting such variability are linked to neuroanatomical and functional changes in prefrontal and temporo‐parietal cortices. Whether these effects are partially mediated by the differences in intra‐cortical myelin remains to be established.

**Method:**

To test this hypothesis, we employed vertex‐wise intra‐cortical myelin maps in *n* = 1,003 people from the Human Connectome Project. Multivariate regression analyses were used to test for the relationship between intra‐cortical myelin and each of the five‐factor model’s personality traits, while accounting for age, sex, intelligence quotient, total intracranial volume, and the remaining personality traits.

**Results:**

Neuroticism *negatively* related to frontal‐pole myelin and *positively* to occipital cortex myelin. Extraversion *positively* related to superior parietal myelin. Openness *negatively* related to anterior cingulate myelin, while Agreeableness *positively* related to orbitofrontal myelin. Conscientiousness *positively* related to frontal‐pole myelin and *negatively* to myelin content in the dorsal anterior cingulate cortex.

**Conclusions:**

Intra‐cortical myelin levels in brain regions with prolonged myelination are *positively* associated with personality traits linked to favorable outcome measures. These findings improve our understanding of the neurobiological underpinnings of variability in common behavioral dispositions.

## 1 INTRODUCTION

Intra‐cortical myelin is an important micro‐structural element of the cortical mantle and is a critical mediator of variability in cognitive, emotional, and behavioral functioning (Grydeland, Walhovd, Tamnes, Westlye, & Fjell, 2013; Nieuwenhuys, 2013; Yasuno et al., 2017). Such effects can depend on the influence that the intra‐cortical myelin content and related cito‐architectural characteristics have on neocortical functioning. For example, Collins and colleagues have argued that the brain areas that have been linked to personality differences (e.g., prefrontal and temporo‐parietal cortices) perform their integrative function via neurons with large dendritic arbors and high synaptic density, two micro‐structural features linked to light intra‐cortical myelin (Collins, Airey, Young, Leitch, & Kaas, 2010). Personality‐related heterogeneity in the intra‐cortical myelin content may thus reflect a significant variation in the computational properties of the neuronal populations in high‐order brain cortices (Collins et al., 2010).

Recently, there has been a growing interest in assessing the content of intra‐cortical myelin via noninvasive but indirect neuroimaging techniques. While other quantifications of intra‐cortical myelin are under development (Alonso‐Ortiz, Levesque, & Pike, 2015; Does, 2018; Heath, Hurley, Johansen‐Berg, & Sampaio‐Baptista, 2018), previous magnetic resonance imaging (MRI) studies have shown that the ratio between the T1‐ and T2‐weighted MRI signal intensity can provide useful information regarding the neocortical myelo‐architecture (Grydeland et al., 2013; Rowley et al., 2017; Shafee, Buckner, & Fischl, 2015). The T1‐weighted and T2‐weighted signals are the two basic MRI signals which, respectively, relate to the spin–lattice and spin–spin relaxation time (the spin is the intrinsic rotation of protons while the lattice is their surrounding environment). In T1‐weighted MRI images of the brain, the gray matter typically shows less signal (i.e., it is darker) than the white matter while the opposite is true for the T2‐weighted images. Although detecting intra‐cortical myelin can be challenging, there is robust evidence showing that the MRI maps derived from the ratio between the T1‐ and T2‐weighted images are sensitive to the intra‐cortical myelin levels (Nakamura, Chen, Ontaneda, Fox, & Trapp, 2017). More specifically, an MRI‐histological (i.e., *post mortem*) study in six cadavers of people with multiple sclerosis, a common neurological disorder which can cause independent demyelination in the white and gray matter (Trapp et al., 2018), demonstrated that the T1‐/T2‐weighted ratio was a simple and reliable measure to assess the neocortical level of myelination (Nakamura et al., 2017).

The MRI T1‐/T2‐weighted ratio also consistently estimates the relative changes in intra‐cortical myelin across the lifespan, that is, from childhood throughout adolescence to adulthood and old age (Grydeland et al., 2013; Rowley et al., 2017; Shafee et al., 2015). Variability in the T1‐/T2‐weighted contrast ratio has also been linked to individual differences in cognitive performances (Grydeland et al., 2013). In particular, the T1/T2‐weighted contrast relates to the differences in *intra*‐individual performance during an attentional task (i.e., the flanker paradigm), indicating that intra‐cortical myelin is *positively* linked to *within*‐subjects variability in cognitive functioning (Grydeland et al., 2013).

The T1/T2‐weighted MRI measure is thus a promising candidate to study behavioral differences *across* individuals. There is robust evidence that evolutionarily more recent brain regions (i.e., the prefrontal and tempo‐parietal cortices) have lighter myelination relative to their sensory‐motor counterparts (Collins et al., 2010; Elston, 2003; Elston, Benavides‐Piccione, & DeFelipe, 2001; Fjell et al., 2015; Hill et al., 2010). This important difference reflects the more complex cyto‐architecture of the former areas in terms of the underlying micro‐structure (e.g., dendritic arbors and spine density) (Collins et al., 2010; Elston, 2003; Elston et al., 2001; Fjell et al., 2015; Hill et al., 2010).

The main aim of this study is to explore the interesting but as yet unaddressed question of how *inter*‐individual differences in personality traits relate to the intra‐cortical myelin content, especially in those brain regions which are known to mediate individual differences in personality traits (i.e., prefrontal and temporo‐parietal cortices) (Beaty et al., 2016; Bjornebekk et al., 2013; Dubois, Galdi, Han, Paul, & Adolphs, 2018; Holmes et al., 2012; Kapogiannis, Sutin, Davatzikos, Costa, & Resnick, 2012; Markett, Montag, & Reuter, undefined/ed; Passamonti et al., 2015; Riccelli, Toschi, Nigro, Terracciano, & Passamonti, 2017; Toschi, Riccelli, Indovina, Terracciano, & Passamonti, 2018; Vartanian et al., 2018). Investigating the link between intra‐cortical myelin and personality traits can also inform existing neurobiological theories of personality that have emphasized the importance of evolutionary and developmental factors in determining the variability of human behavior (DeYoung, 2015; Durbin et al., 2016; Johnson et al., 2000; Riley, Peterson, & Smith, 2017; Roberts & Mroczek, 2008). For example, epidemiological and psycho‐sociological studies in large cohorts of people across the life span have found that some personality profiles relate to more “mature” behavioral patterns in terms of emotional (low Neuroticism), cognitive (high Conscientiousness), and social (high Agreeableness) functioning (Blonigen, Carlson, Hicks, Krueger, & Iacono, 2008; Donnellan, Conger, & Burzette, 2007; Roberts, Caspi, & Moffitt, 2001; Terracciano, Yannick, Luchetti, & Sutin, in press). The presence of such emotional, cognitive, and social stability has important consequences in terms of psychological and well‐being outcome measures including life satisfaction, academic/professional achievement or general health, longevity, and risk to develop dementia (Noftle & Robins, 2007; Ozer & Benet‐Martinez, 2006; Roberts, Lejuez, Krueger, Richards, & Hill, 2014; Sutin et al., 2016). Nevertheless, it remains unclear which are the neurobiological underpinnings of this behavioral stability and in particular whether they are dependent on the differences in intra‐cortical myelin and myelination.

Thus far, research in personality neuroscience has made important progresses in understanding the neurological basis of individual differences in cognitive, emotion, and behavioral dispositions (Corr, 2006; Corr & Mobbs, 2018). However, even studies with large samples of participants have almost focused on canonical brain imaging measures including gray matter density, white matter integrity, and brain function, either at the level of single regions or, more recently, using “connectomic” or large‐scale network approaches (Beaty et al., 2016; Bjornebekk et al., 2013; Dubois et al., 2018; Holmes et al., 2012; Kapogiannis et al., 2012; Markett et al., undefined/ed; Passamonti et al., 2015; Riccelli et al., 2017; Toschi et al., 2018; Vartanian et al., 2018). Despite such progresses in understanding the neuroanatomical and functional basis of personality, it is still undetermined whether measures of intra‐cortical myelin, which are hypothesized to reflect differences in brain growth, maturation, and functioning (Grydeland et al., 2013; Nieuwenhuys, 2013; Yasuno et al., 2017), can represent a valid biomarker of individual differences in personality.

Currently, one study with *n* = 37 participants has attempted to characterize how intra‐cortical myelin relates to personality differences (Yasuno et al., 2017). Our study aimed at expanding the preliminary findings from this earlier study and at exploring, in a large, homogenous and well‐characterized sample of individuals (*n *= 1,003), how the intra‐cortical myelin content relates to individual differences in personality traits.

We predicted that the associations between intra‐cortical myelin and personality traits were regionally specific and localized to those brain areas that have been consistently implicated in high‐level socio‐affective functioning (i.e., prefrontal and temporo‐parietal cortices) (Beaty et al., 2016; Bjornebekk et al., 2013; Dubois et al., 2018; Holmes et al., 2012; Kapogiannis et al., 2012; Riccelli et al., 2017; Rueter, Abram, MacDonald, Rustichini, & DeYoung, 2018; Toschi et al., 2018; Vartanian et al., 2018; Tompson, Falk, Vettel, & Bassett, 2018). The prefrontal and temporo‐parietal cortices express prolonged myelination during development and have lighter myelin content relative to their sensory‐motor counterparts (McGaugh, Weinberger, & Lynch, 1995; Leipsic, 1901; Miller et al., 2012).

## 2 PARTICIPANTS and METHODS

The sample of participants (*n* = 1,003 individuals) in this study was drawn from the Human Connectome Project (HCP), a large international project that has provided access to a set of high‐quality behavioral and neuroimaging measures (Van Essen et al., 2013) (https://www.humanconnectome.org/). The demographic and other variables of the HCP sample are summarized in Table 1 while the personality data are reported in Table 2.

**Table 1:** Demographic and clinical characteristics of the sample included in the study (*n = *1,003 participants, 550 females)

	1st quartile	Median	3rd quartile
Age (years)	26	29	32
Education (years)	14	16	16
Height (cm)	165	170	178
Weight (kg)	64	75	89
Body mass index	23	25	29
Systolic blood pressure (mmHg)	114	123	132
Diastolic blood pressure (mmHg)	70	76	83
Conduct problems during childhood	0	0	1
Panic disorder symptoms	0	0	0
Depressive symptoms	0	0	0
Cigarettes per week	0	0	0
Drinks per week	0	2	7
Race (%)	Asian/Natural Hawaiian/Other Pacific Islands: 5.5%		
	Black or African American: 15.6%		
	White: 74.7%		
	More than one: 2.6%		
	Unknown or not reported: 1.6%		
Ethnicity (%)	Hispanic/Latino: 8.6%		
	Not Hispanic/Latino: 90.4%		
	Unknown or not reported: 1.0%		
Handedness (%)	Right handed: 83.6%		
	Left handed: 7.5%		
	Mixed: 8.9%		

**Table 2:** Personality data as assessed via the NEO‐five factor inventory questionnaire (*n* = 1,003 participants, 550 females)

			Range	
	Mean	*SD*	Min.	Max.
Neuroticism	16.5	7.3	0	43
Extraversion	30.6	6.0	10	47
Openness	28.2	6.1	10	45
Agreeableness	33.5	5.8	10	48
Conscientiousness	34.5	5.9	12	48

Note

For each personality trait, the mean, standard deviation (*SD*) as well as minimum and maximum values are reported.

In brief, all participants were young adults (54.8% females; mean‐age: 29 years) with no major medical conditions including obesity, hypertension, alcohol misuse, anxiety, depression, or other psychiatric or neurologic disorders as well as no history of behavioral problems during childhood (e.g., conduct disorder). The majority of the participants were right‐handed White Americans with a non‐Hispanic or Latinos background. The primary participant pool, initially selected through screening interviews, comes from healthy individuals born in Missouri, based on the data from the Missouri Department of Health and Senior Services Bureau of Vital Records. The concept of “healthy” was defined aiming for a pool that is representative of the population at large in order to capture a wide range of variability in healthy individuals with respect to behavioral, ethnical, and socioeconomic diversity. Extensive additional details are provided in Van Essen et al. (2013).

### 2.1 Personality assessment

The five‐factor model (FFM) personality traits were assessed via the NEO Five‐Factor Inventory (NEO‐FFI) (Costa & McCrae, 1992; Terracciano, 2003). The NEO‐FFI is composed by 60 items, 12 for each of the five factors. For each item, participants reported their level of agreement on a 5‐point Likert scale, from strongly disagree to strongly agree. The NEO instruments have been previously validated in the USA and several other countries (McCrae & Terracciano, 2005). The recently discovered bug in the scoring of HCP Agreeableness data was corrected prior to any further processing (personal communication on HCP mailing list on 03/09/2018 20:48 CEST).

### 2.2 MRI scanning protocol

Subjects were scanned at the Washington University in St. Louis and at the Northwestern University on Siemens 3T Tim Trios using a 12‐channel head coil. A 3D T1w magnetization‐prepared rapid gradient echo sequence was acquired (MPRAGE; TR = 2,400 ms, TE = 2.14 ms, TI = 10,009 ms, 8° flip angle, bandwidth = 210 Hz/pixel, echo spacing = 7.6 ms, FOV 224 × 224 × 178 mm, matrix 320 × 320, 256 slices, 0.7 mm isotropic resolution). A generalized auto‐calibrating partially parallel acquisition (GRAPPA) factor of 2 in combination with 50% phase oversampling (acquisition time 7:40 min) gave a signal‐to‐noise ratio intermediate to that with no parallel imaging and that with a GRAPPA factor of 2 and no phase oversampling. A 3D T2w sampling perfection with application of an optimized contrast using a different angle evolutions sequence was acquired (SPACE; TR = 3,200 ms, TE = 565 ms, variable flip angle, bandwidth = 744 Hz/pixel, echo spacing = 3.53 ms, matrix 320 × 320, 256 slices, 0.7‐mm isotropic resolution). A GRAPPA factor of 2 was used with no phase oversampling (acquisition time of 8:24 min). Both scans were acquired sagittally. Total in‐scanner time is divided up in two consecutive sessions (day 1 and day 2) in which different modalities (e.g., structural, functional, diffusion) are acquired, always in the same order. Session 1 is preceded by a mock scanner session in order for the subjects to acclimatize to the scanner environment.

### 2.3 Image processing

Preprocessed cortical myelin maps were downloaded from the HCP consortium database (https://db.humanconnectome.org/). Such myelin maps were generated from T1‐weighted and T2‐weighted contrasts as described in detail by the HCP consortium (Glasser & Van Essen, 2011). Accordingly, T1‐weighted and T2‐weighted volumes used for cortical myelin map estimation had been preprocessed according to the standard, state‐of‐the‐art HCP pipeline (Glasser et al., 2013), which can also be found online (https://www.humanconnectome.org/storage/app/media/documentation/s1200/HCP_S1200_Release_Reference_Manual.pdf). This pipeline uses FreeSurfer to generate white, pial, and mid‐thickness surfaces (Dale, Fischl, & Sereno, 1999; Fischl, Sereno, Tootell, & Dale, 1999; Segonne et al., 2004; Sled, Zijdenbos, & Evans, 1998), which are then mapped to the 164 k vertex fs_LR mesh using caret and the Connectome workbench.

Within preprocessing, the T2‐weighted image is registered to the T1‐weighted image using FSL’s FLIRT by applying a rigid body transformation and using mutual information as cost function. The T2‐weighted image is successively resampled with FSL’s applywarp tool to guarantee the overlap with the T1‐weighted image. It is interesting to note how, in this context, taking the ratio of the two contrasts increases the sensitivity to intra‐cortical myelin and simultaneously decreases bias. Given that the contrast due to myelin content (*m*) is approximately proportional to the intensity in a T1‐weighted image and approximately inversely proportional to the intensity (1/*m*) in a T2‐weighted image, while the receive bias field can be represented by (say) *b* in both the images, by taking the *T1‐weighted/T2‐weighted* ratio the generated contrast will be approximately proportional to *b* ^2^, that is, we will have an enhanced myelin contrast, while cancelling out most of the bias field. In other words (see also [Glasser & Van Essen, 2011]),

$$\frac{T_{1}w}{T_{2}w}∼\frac{\mathrm{mb}}{1/mb}=m^{2}$$

Furthermore, given that T1‐weighted and T2‐weighted images are affected by uncorrelated noise, taking their ratio also results in an increased myelin contrast relative to noise (i.e., increased contrast‐to‐noise ratio).

### 2.4 Statistical analyses

To perform vertex‐wise analyses, cortical myelin maps for all subjects were converted to freesurfer “fsaverage” space (a 164k vertex space which represents a standard‐subject, common space surface reconstruction template) for statistical inference using the Connectome workbench (https://wiki.humanconnectome.org/download/attachments/63078513/Resampling-FreeSurfer-HCP.pdf). Next, we investigated the associations between subject‐specific intra‐cortical myelin measures at each vertex and individual scores in all FFM personality scores by formulating a multivariate general linear model. This was done to assess the independent effect of each FFM trait on the intra‐cortical myelin content while factoring out any possible confounding effect driven by the remaining personality factors. The regression models also included age, total intracranial volume, intelligence quotient, and sex as covariates of no interest. To control for false positives as well as multiple comparisons, cluster correction was completed using Monte Carlo simulation (with a vertex‐wise cluster forming the threshold of *p* < 0.001) at a cluster‐wise P (CWP) value of 0.05. This entails: (a) synthesizing a z‐map, (b) smoothing of the z‐map, (c) thresholding at the chosen level (see above), (d) finding clusters in the thresholded z‐map, and (e) recording the area of the largest cluster. Steps 1–5 are repeated n times (*n* = 10,000 here), giving rise to an *n*‐sample distribution estimate of the maximum cluster size under the null hypothesis. Successively, for each cluster found after thresholding of the original data, a *p*‐value is assigned which corresponds to the probability of seeing a cluster of that size or larger during simulation. This procedure is described in Hagler, Saygin, and Sereno (2006).

We also calculated the maps of the effect sizes for statistically significant findings by deriving partial correlation coefficients directly from the general linear model fit for each regressor/contrast.

## 3 RESULTS

### 3.1 Behavioral results

A summary of the demographic and other behavioral characteristics of the sample is reported in Table 1. The mean FFM personality scores, their standard deviation as well as maximum and minimum values are reported in Table 2.

### 3.2 Median intra‐cortical myelin map independently of personality differences

**Figure 1:**
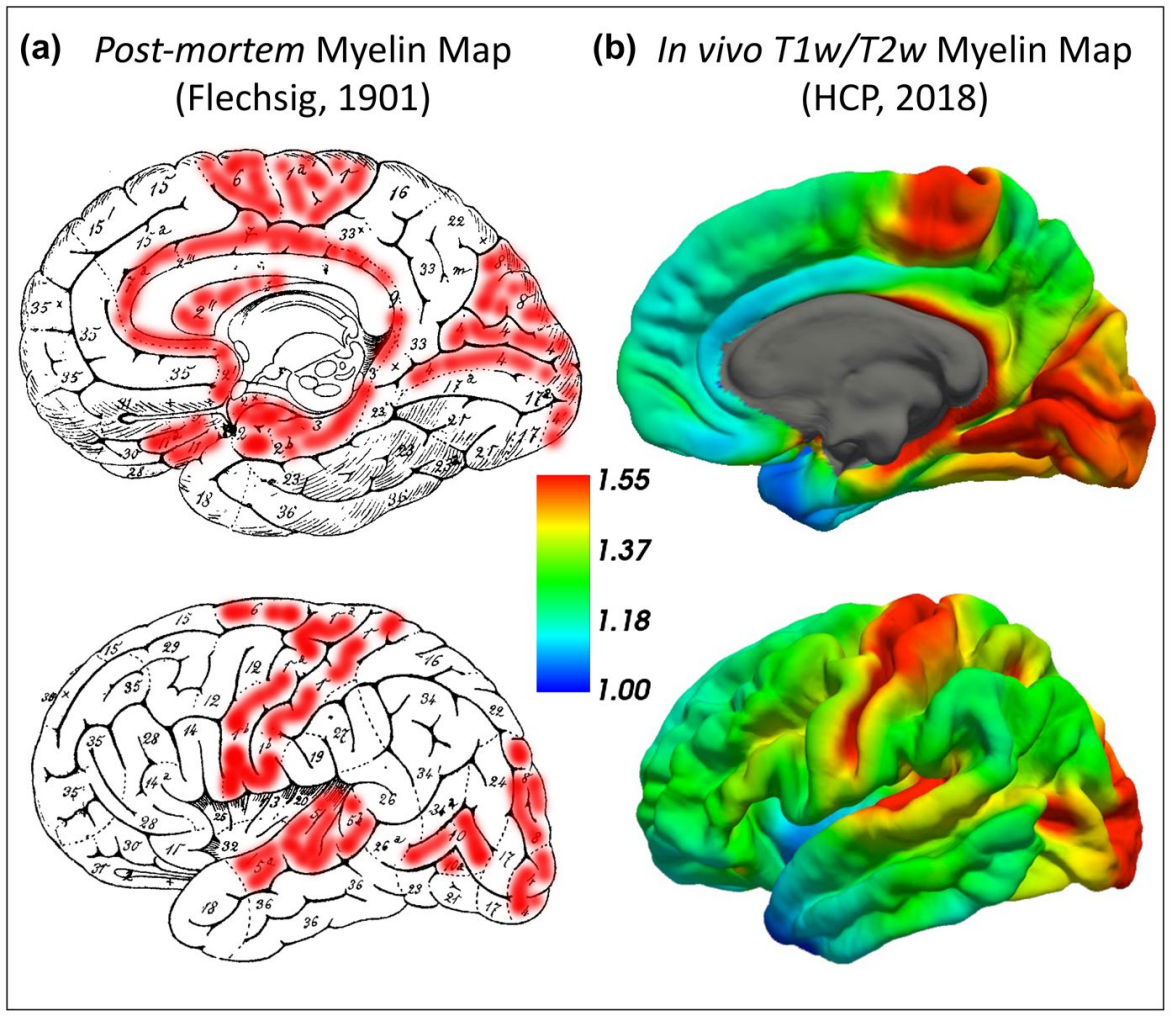
(a) Flechsig’s *postmortem* myelination map (modified from Leipsic, 1901). The number indicates the progressive order of myelination and the extent of the intra‐cortical myelin content, with higher numbers representing later myelination during development and consequently lighter local myelin content. The regions shaded in red are the brain regions with earlier myelination and higher intra‐cortical myelin content. These areas strikingly resemble the regions shown in red in B, which are those where higher T1/T2‐weighted signal was identified. (b) In vivo myelo‐architectonic map based on the T1‐/T2‐weighted signal intensity ratio in *n = *1,003 participants independently of personality differences. The color bar represents the brain regions with high (red) and low (blue) intra‐cortical myelin content (median values). HCP, Human Connectome Project

The overall median intra‐cortical myelin map in *n* = 1,003 individuals (i.e., independently of personality differences) was highly consistent with previously published data using T1/T2‐weighted ratio measures in a much smaller part of the same HCP sample (*n* = 69) (Figure 1) (Glasser & Van Essen, 2011). Importantly, these findings are also in keeping with the myelo‐architectonic map originally developed by Paul Flechsig over a century ago (Figure 1) (Leipsic, 1901). In Flechsig’s histological (i.e., *post‐mortem*) map, the number associated with each region reflects the relative order of myelination during development, with higher numbers (from 10 onwards) indicating later myelination (Figure 1). Of note, the regions with later myelination (i.e., from number 10 onwards) are also the brain region with lighter myelin content (Figure 1) (Leipsic, 1901).

### 3.3 Intra‐cortical myelin content in relation to each of the FFM personality traits

#### 3.3.1 Neuroticism

**Figure 2:**
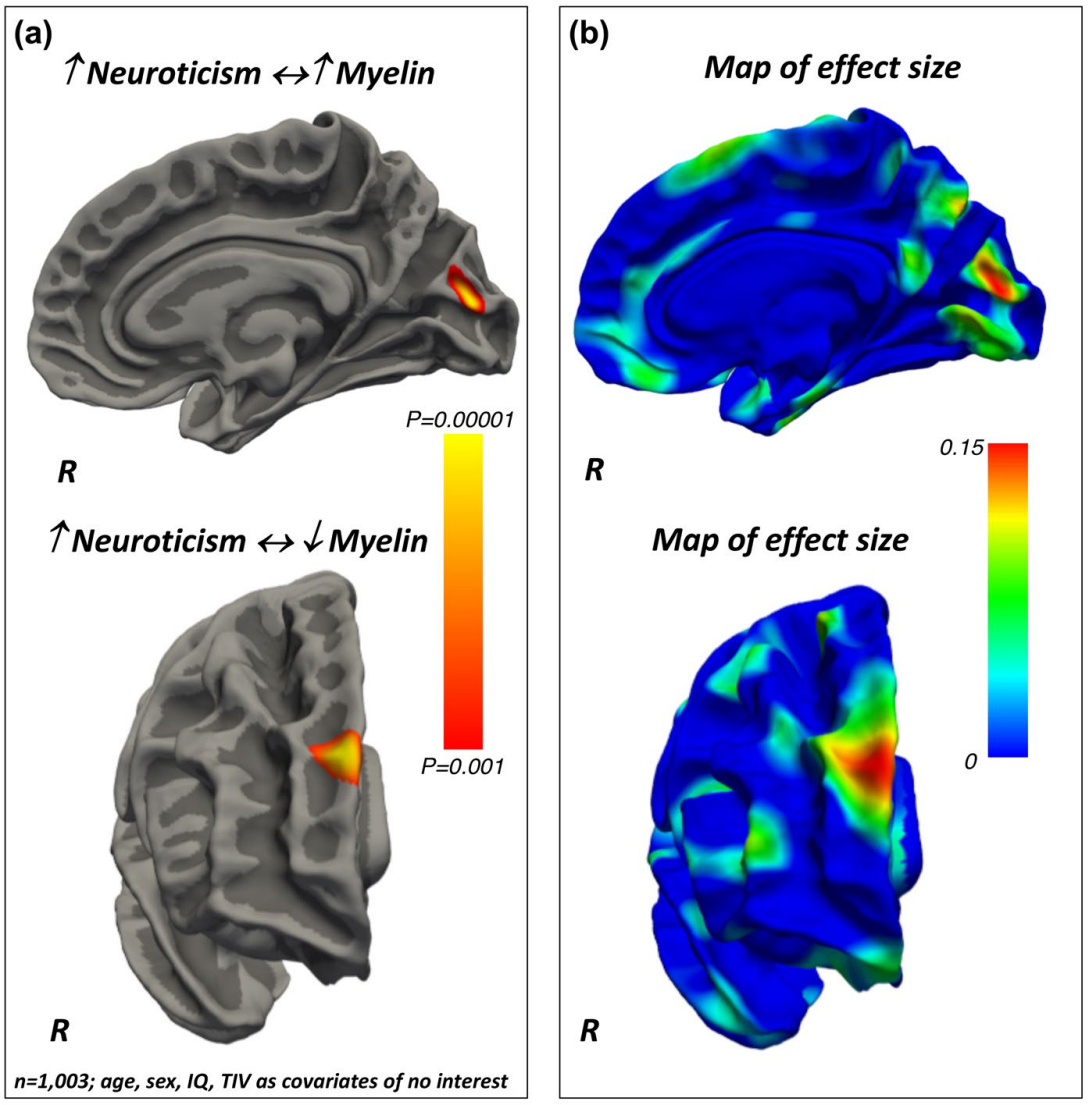
(a) Positive and negative associations between Neuroticism and regionally specific intra‐cortical myelin content. The color bar represents the *p*‐values for the associations. (b) Maps of the effect sizes for the findings presented in panel (a). The color bar represents the strength of the effect sizes. IQ, intelligence quotient; TIV, total intracranial volume

Neuroticism *positively* related to the intra‐cortical myelin content in the occipital cortex (Brodmann’s area 18/19) (Figure 2, Table 3). At the same time, a significantly *negative* correlation was found between Neuroticism and the intra‐cortical myelin levels in the prefrontal cortex (PFC) pole (Brodmann’s area 10) (Figure 2, Table 3).

**Table 3:** Vertex‐wise results as a function of Neuroticism scores (*n* = 1,003 participants, 550 females)

	NEUROTICISM						
Region (Brodmann’s area, BA)	Hemisphere	Max	Size (mm^2^)	*X*	*Y*	*Z*	CWP
	*Positive associations*						
Para‐Striate Visual Area (BA18)	R	4.2	216.1	4	−83	12	0.040
	*Negative association*						
Fronto‐Polar Prefrontal Cortex (BA10)	R	3.5	260.2	13	61	28	0.023

Note

Positive and negative associations between Neuroticism scores and intra‐cortical myelin levels. *X*, *Y*, *Z*: Montreal Neurological Institute (MNI) coordinates of the local maxima; R right hemisphere; Max: the maximum –log_10_ of the cluster‐wise *p*‐value (CWP).

#### 3.3.2 Extraversion

**Figure 3:**
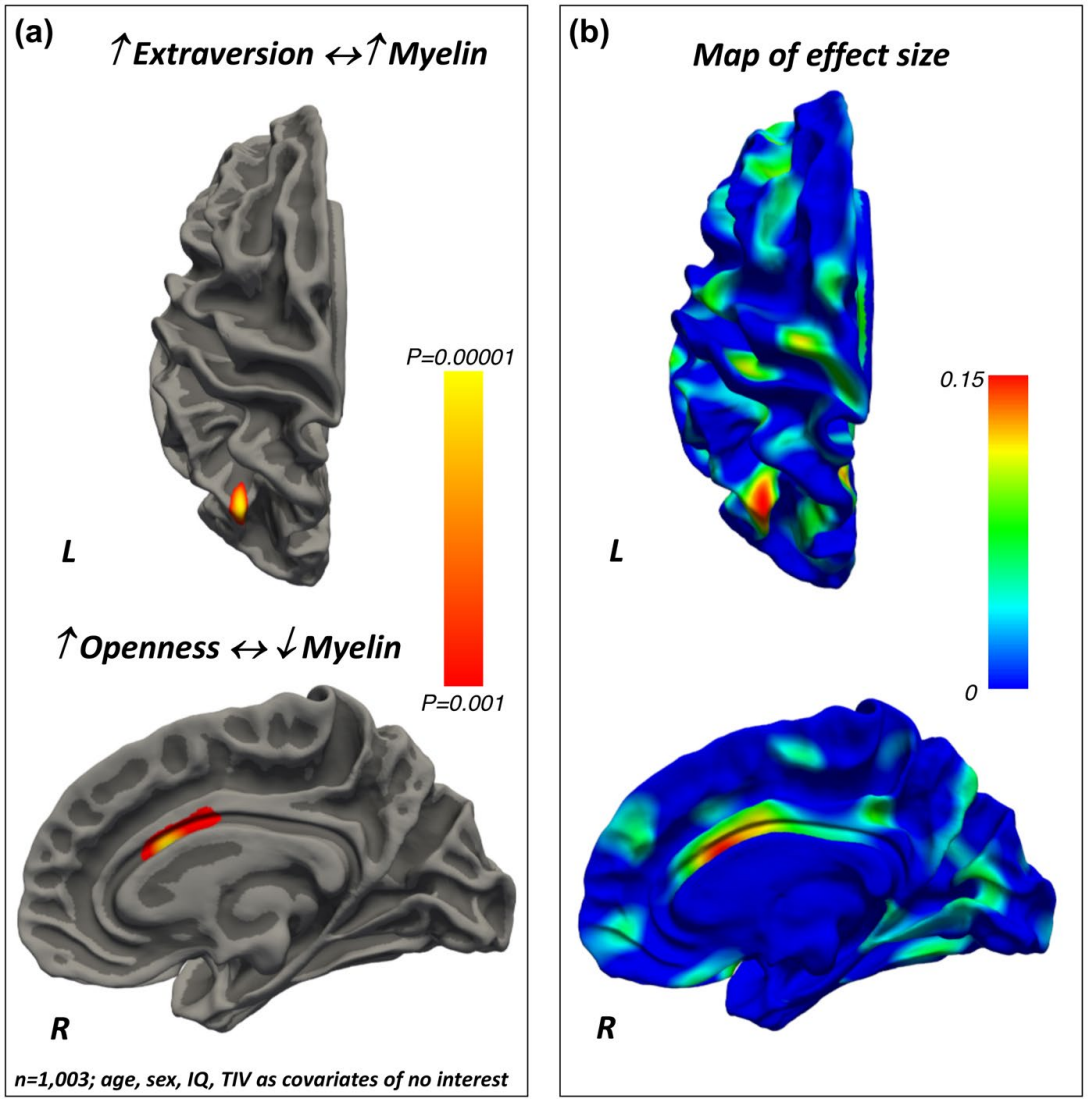
(a) Positive and negative associations between (respectively) Extraversion and Openness and local intra‐cortical myelin content. The color bar represents the *p‐*values for the associations. (b) Maps of the effect sizes for the findings are presented in panel (a). The color bar represents the strength of the effect sizes. IQ, intelligence quotient; TIV, total intracranial volume

Extraversion was *positively* associated with the intra‐cortical myelin content in the superior parietal lobule (Brodmann’s area 7) (Figure 3, Table 4).

**Table 4:** Vertex‐wise results as a function of Extraversion scores (*n* = 1,003 participants, 550 females)

	EXTRAVERSION						
Region (Brodmann’s area, BA)	Hemisphere	Max	Size (mm^2^)	*X*	*Y*	*Z*	CWP
	*Positive association*						
Superior Parietal Lobule (BA7)	L	3.7	222.4	−33	−69	47	0.036

Note

Positive association between Extraversion scores and intra‐cortical myelin levels. L left hemisphere; *X*, *Y*, *Z*: Montreal Neurological Institute (MNI) coordinates of the local maxima; L: left hemisphere; Max: the maximum –log_10_ of the cluster‐wise *p*‐value (CWP).

#### 3.3.3 Openness

Openness was *negatively* associated with the intra‐cortical myelin content in the anterior cingulate cortex (Figure 3, Table 5).

**Table 5:** Vertex‐wise results as a function of Openness scores (*n* = 1,003 participants, 550 females)

	OPENNESS						
Region (Brodmann’s area, BA)	Hemisphere	Max	Size (mm^2^)	*X*	*Y*	*Z*	CWP
	*Negative association*						
Dorsal Anterior Cingulate Cortex (BA32)	R	4.7	213.2	3	15	24	0.042

Note

Negative association between Openness to Experience scores and intra‐cortical myelin levels. *X*, *Y*, *Z*: Montreal Neurological Institute (MNI) coordinates of the local maxima; R right hemisphere; Max: the maximum –log_10_ of the cluster‐wise *p*‐value (CWP).

#### 3.3.4 Agreeableness

**Figure 4:**
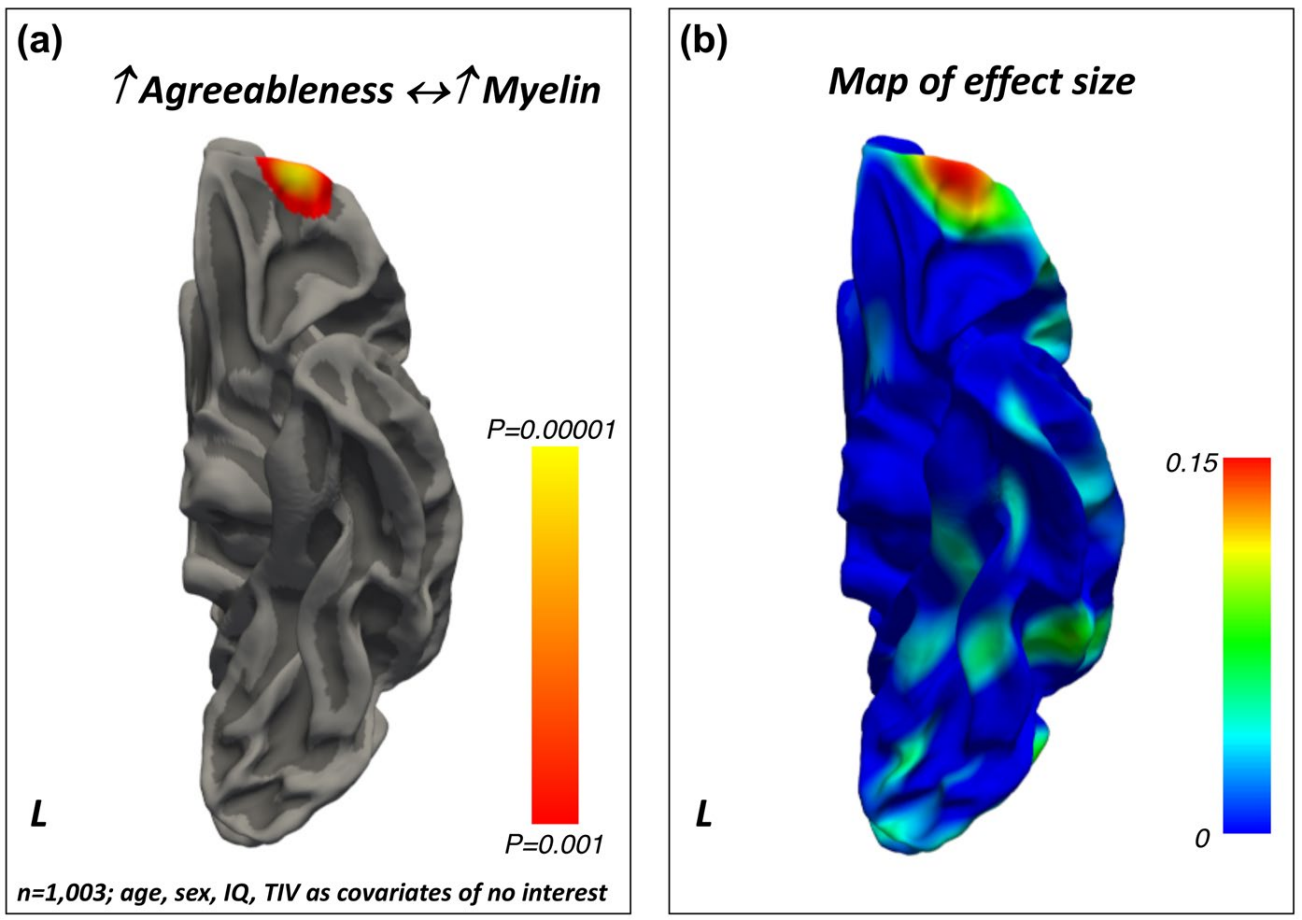
(a) Positive association between Agreeableness and regionally specific intra‐cortical myelin content. The color bar represents the *p‐*values for the association. (b) Maps of the effect sizes for the findings presented in panel (a). The color bar represents the strength of the effect sizes. IQ, intelligence quotient; TIV, total intracranial volume

A *positive* relationship was found between Agreeableness and the intra‐cortical myelin content in the anterior orbitofrontal cortex (Brodmann’s area 11) (Figure 4, Table 6).

**Table 6:** Vertex‐wise results as a function of Agreeableness scores (*n* = 1,003 participants, 550 females)

	AGREEABLENESS						
Region (Brodmann’s area, BA)	Hemisphere	Max	Size (mm^2^)	*X*	*Y*	*Z*	CWP
	*Positive associations*						
Anterior Orbitofrontal Cortex (BA11)	L	4.8	508.1	−27	57	−14	0.0004

Note

Positive association between Agreeableness scores and intra‐cortical myelin levels. *X*, *Y*, *Z*: Montreal Neurological Institute (MNI) coordinates of the local maxima; L: left hemisphere; Max: the maximum –log_10_ of the cluster‐wise *p*‐value (CWP).

#### 3.3.5 Conscientiousness

**Figure 5:**
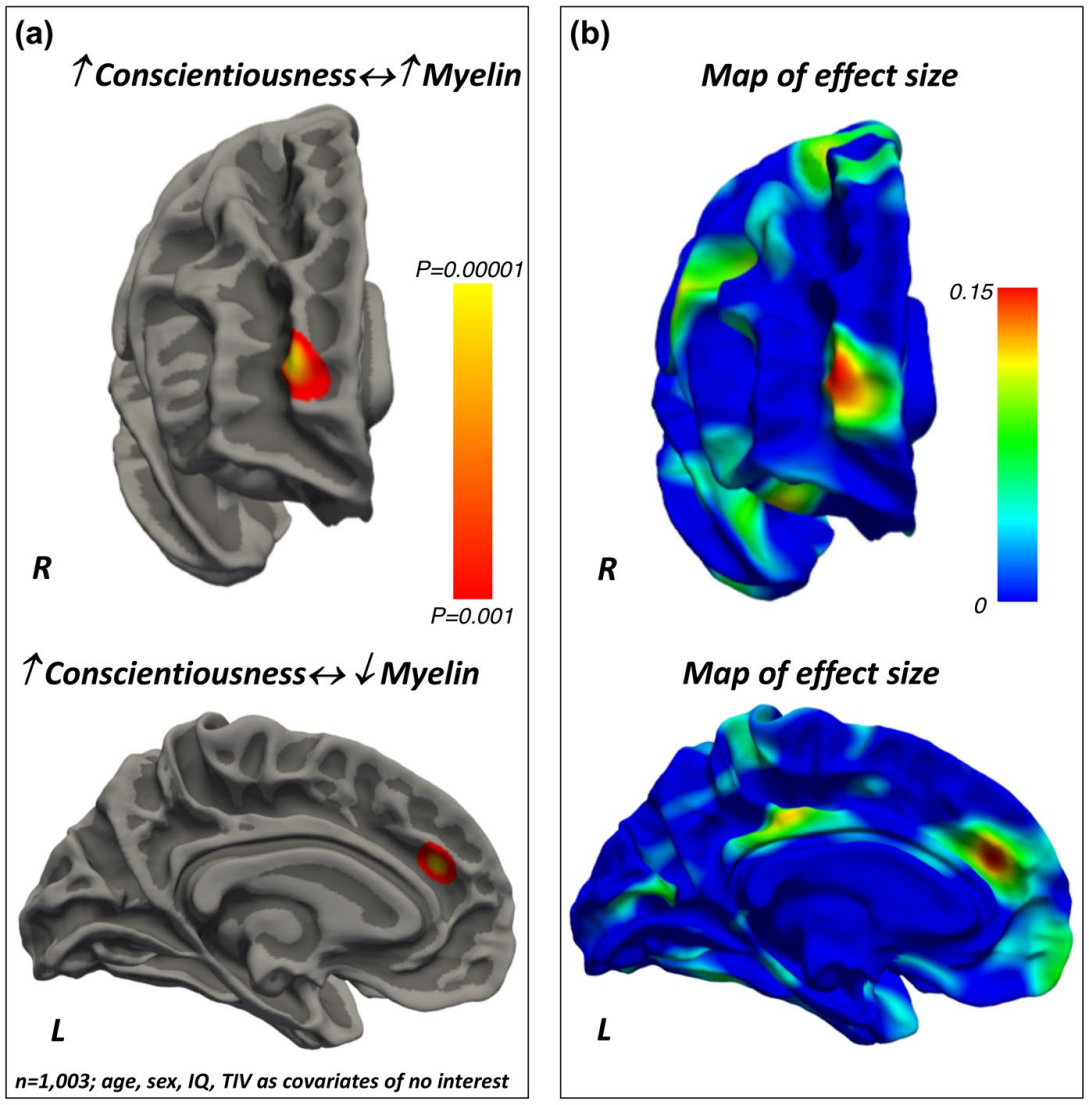
(a) Positive and negative associations between Conscientiousness and local intra‐cortical myelin content. The color bar represents the *p*‐values for the association. (b) Maps of the effect sizes for the findings presented in panel (a). The color bar represents the strength of the effect sizes. IQ, intelligence quotient; TIV, total intracranial volume

Conscientiousness *positively* related to the intra‐cortical myelin content in the PFC pole (Brodmann’s area 10) and *negatively* to the intra‐cortical myelin levels in the dorsal anterior cingulate cortex (Brodmann’s area 32) (Figure 5, Tables 7).

**Table 7:** Vertex‐wise results as a function of Conscientiousness scores (*n* = 1,003 participants, 550 females)

	COSCIENTIOUSNESS						
Region (Brodmann’s area, BA)	Hemisphere	Max	Size (mm^2^)	*X*	*Y*	*Z*	CWP
	*Positive associations*						
Fronto‐Polar Prefrontal Cortex (BA10)	R	4.8	364.7	26	62	13	0.004
	*Negative association*						
Dorsal Anterior Cingulate Cortex (BA32)	L	4.9	225.1	−9	38	22	0.035

Note

Positive and negative associations between Conscientiousness scores and intra‐cortical myelin levels. *X*, *Y*, *Z*: Montreal Neurological Institute (MNI) coordinates of the local maxima; L: left hemisphere; R right hemisphere; Max: the maximum –log_10_ of the cluster‐wise *p*‐value (CWP)

## 4 DISCUSSION

Our findings demonstrate that intra‐cortical myelin content relates to personality differences, as assessed via the FFM taxonomy. Specifically, Neuroticism related *positively* to intra‐cortical myelin in the visual cortex and *negatively* to myelin content in the PFC pole. Extraversion related *positively* to myelin levels in the superior parietal cortex, while a *negative* association was found between the myelin content in the anterior cingulate and Openness. Agreeableness related *positively* to myelin levels in the orbitofrontal cortex. Conscientiousness related *positively* to the intra‐cortical myelin content in the prefrontal pole and *negatively* with the myelin content in the dorsal anterior cingulate cortex. The results for each of the FFM traits were obtained using multivariate statistical models that controlled for the remaining FFM traits as well as for age, sex, intelligence quotient, and total intra‐cranial volume variability. Together, these findings show that intra‐cortical myelin, as estimated via the MRI‐based T1/T2‐weighted contrast ratio, is a sensitive measure to investigate the neuroanatomical basis of the behavioral differences described by the FFM of personality.

Recently, another study has linked the intra‐cortical myelin measures derived from the T1/T2‐w ratio and the FFM of personality and has found a negative association between Openness and myelin content in a series of brain regions including the medial frontal cortex, anterior/posterior cingulate cortex, and posterior insula (Yasuno et al., 2017). Although consistent with some of our findings, it is difficult to directly compare the results of this earlier study with the current data due to significant differences in the sample size (*n* = 37 vs. *n* = 1,003 participants), and MRI parameters (repetition‐time, echo‐time, number of slices, and voxel size, for both the T1 and T2 MRI sequences).

At the group level (i.e., regardless of personality differences), we replicated the previous findings obtained in approximately 7% (*n* = 69) of the same sample of participants, which were in turn consistent with the original *postmortem* map of myelination and myelin content provided by Paul Flechsig over a century ago (Arshad, Stanley, & Raz, 2017; Ganzetti, Wenderoth, & Mantini, 2014; Glasser & Van Essen, 2011; Leipsic, 1901; Shafee et al., 2015). Together, the data showed that the sensory‐motor cortices display the highest levels of myelin and the earliest myelination during brain maturation (Arshad et al., 2017; Ganzetti et al., 2014; Glasser & Van Essen, 2011; Leipsic, 1901; Shafee et al., 2015). Conversely, associative regions such as the prefrontal and temporo‐parietal cortices tend to myelinate later on during development and consequently show lighter myelin content compared to their sensory‐motor counterparts (Arshad et al., 2017; Ganzetti et al., 2014; Glasser & Van Essen, 2011; Leipsic, 1901; Shafee et al., 2015). A similar relationship has been described between the intra‐cortical myelin levels and the degree of cortical expansion during evolution (Miller et al., 2012). Relative to other great apes, the size of lightly myelinated brain regions in humans has expanded more than the size of heavily myelinated areas (Miller et al., 2012). On the other hand, chimpanzees have more rapid myelination than human beings, especially in associative areas such as the prefrontal and temporo‐parietal cortices (Miller et al., 2012). These ontogenetic and phylogenetic processes can help explain our current findings, which can also be interpreted in the context of the theoretical frameworks that have emphasized the importance of development and evolutionary factors in determining personality differences.

The personality‐related variability in the intra‐cortical myelin content can thus represent an important proxy measure of the underlying neurodevelopmental mechanisms that shape subject‐specific attitudes in cognitive, emotional, and behavioral functions. For instance, the fact that Neuroticism scores related *negatively* to myelin content in the prefrontal pole may result from a delayed maturation of a region that is expected to have relatively light myelin content and prolonged myelination (Collins et al., 2010; Elston, 2003; Elston et al., 2001; Fjell et al., 2015; Hill et al., 2010). In other words, a reduced intra‐cortical myelin content that we identified cross‐sectionally in people with high Neuroticism scores may be explained by a delayed or altered trajectory in the development or accumulation of myelin (myelination process), although to confirm this hypothesis longitudinal studies are needed.

Differences in the intra‐cortical myelin content can also reflect the variability in the cito‐architectural features such as dendritic ramification and synaptic density which in turn have a strong impact on neuronal functioning (Collins et al., 2010). Reduced intra‐cortical myelin in the PFC of people scoring high in Neuroticism is also consistent with recent findings from people with major depression disorders (MDD) (Sacchet & Gotlib, 2017). The study by Sacchet and colleagues used quantitative MRI to assess myelin content in *n* = 40 people with MDD and *n* = 40 controls and found that the MDD participants have overall lower levels of myelin than controls, and that myelin in the PFC was reduced in people with MDD and more frequent episodes of depression (Sacchet & Gotlib, 2017). Such reduced intra‐cortical myelin in the PFC of people with high levels of Neuroticism and MDD may represent an intermediate phenotypic expression of the executive dysfunctions and/or problems in regulating emotions that have been described in these individuals (Harenski, Kim, & Hamann, 2009; Levesque et al., 2003; Ochsner & Gross, 2005; Phillips, Ladouceur, & Drevets, 2008). However, it remains to be elucidated why Neuroticism also *positively* related to intra‐cortical myelin in brain areas with relatively high myelin content and rapid myelination rate (i.e., the visual cortex) (Collins et al., 2010; Elston, 2003; Elston et al., 2001; Fjell et al., 2015; Hill et al., 2010).

Interestingly, Agreeableness was *positively* linked to myelin content in the orbitofrontal cortex, a brain region that tends to myelinate late and that has been repeatedly associated with temperamental attitudes related to social behavior both in human beings and their phylogenetic ancestors (Hare & Kwetuenda, 2010; Palagi, 2006; Rilling et al., 2011).

Conscientiousness *positively* related to myelin levels in the PFC pole (an area with late myelination) and *negatively related* to the dorsal anterior cingulate cortex (an area myelinating earlier), while Openness was *negatively *associated with myelin content in the anterior cingulate cortex. These regional effects are in keeping with recent studies which have used measures of brain structure and function and linked the dorsal anterior cingulate cortex and the “goal priority network” with differences in Conscientiousness (Rueter et al., 2018), or the default mode network to Openness (Beaty et al., 2016; Vartanian et al., 2018). Overall, our data indicate that variability in personality traits is mediated by a complex and regional‐specific combination of increased and decreased myelination which in turn may reflect heterogeneity in the underlying dendritic architecture and neuronal density (Collins et al., 2010; Elston, 2003; Elston et al., 2001; Fjell et al., 2015; Hill et al., 2010).

This study also suggests that enhanced intra‐cortical myelin synthesis, which is driven by genetic factors and/or complex gene by environment interactions, may be a key determinant of improved behavioral outcomes associated with low Neuroticism, high Extraversion, high Agreeableness, and high Conscientiousness. Examples of these outcome measures include indices of well‐being, occupational/educational achievement, risk to develop dementia, and longevity (Kern & Friedman, 2008; Sutin, Luchetti, Stephan, Robins, & Terracciano, 2017; Terracciano, An, Sutin, Thambisetty, & Resnick, 2017).

### 4.1 Strengths and limitations

Our study has two main strengths: (a) it uses standardized vertex‐wise analyses to assess the relationships between the intra‐cortical myelin content and personality traits, and (b) it employs a large and well‐characterized sample of participants in terms of personality and demographic features (*n* = 1,003 people). Regarding its potential shortcomings, it is possible that errors in the surface reconstruction might have affected the estimation of the myelin content in heavily myelinated regions such as the primary visual cortex, although we were reassured by the fact that the results at the group level (i.e., independently of personality differences) were highly consistent with the histological maps published by Paul Flechsig over a century ago (Leipsic, 1901). As many other studies in the field, our work was based on self‐report measures of personality which inevitably depend on people’s judgment on their own behavior. Although future research should examine the impact of alternative sources of information (e.g., reports by friends, relatives, etc.), the high validity and reproducibility of the FFM scores, particularly in healthy and young adults, is well established (Young & Schinka, 2001). Finally, we acknowledge the cross‐sectional nature of this study and the necessity to run longitudinal research to answer the important question of how the developmental trajectories of intra‐cortical myelination relate to differences in personality traits.

### 4.2 Summary and conclusions

In conclusion, our results showed that intra‐cortical myelin is significantly linked to variability in personality traits. Of note, most of the effects were localized in high‐order brain regions, a group of cortical areas with light myelin content and prolonged myelination, both at the phylogenetic and the ontogenetic levels. This may depend on the fact that many of the FFM personality traits relate to high‐level cognitive and socio‐affective skills which have significantly evolved in human beings and that are critically mediated by the cito‐architectonically complex and lightly myelinated cortices.

Finally, the statistically robust relation between heterogeneity in the intra‐cortical myelin content and personality differences in healthy people suggests that the myelo‐architectural features may show even more pronounced changes in people with psychiatric illnesses such as major depressive disorders.

## CONFLICT OF INTERESTS

The authors declare no potential conflicts of interest with respect to the research, authorship, and/or publication of this article.
